# A Genomics Based Discovery of Secondary Metabolite Biosynthetic Gene Clusters in *Aspergillus ustus*

**DOI:** 10.1371/journal.pone.0116089

**Published:** 2015-02-23

**Authors:** Borui Pi, Dongliang Yu, Fangwei Dai, Xiaoming Song, Congyi Zhu, Hongye Li, Yunsong Yu

**Affiliations:** 1 Department of Infectious Diseases, Sir Run Run Shaw Hospital, College of Medicine, Zhejiang University, Hangzhou, Zhejiang, 310016, China; 2 College of Life and Environmental Sciences, Hangzhou Normal University, Hangzhou, Zhejiang, 310036, China; 3 Center of Experimental Animals, Zhejiang Academy of Medical Sciences, Hangzhou, Zhejiang, 310013, China; 4 Laboratory Animal Centre of Hangzhou Normal University, Hangzhou, Zhejiang, 310036, China; 5 Institute of Biotechnology, Zhejiang University, Hangzhou, Zhejiang, 310058, China; Technical University of Denmark, DENMARK

## Abstract

Secondary metabolites (SMs) produced by *Aspergillus* have been extensively studied for their crucial roles in human health, medicine and industrial production. However, the resulting information is almost exclusively derived from a few model organisms, including *A. nidulans* and *A. fumigatus*, but little is known about rare pathogens. In this study, we performed a genomics based discovery of SM biosynthetic gene clusters in *Aspergillus ustus*, a rare human pathogen. A total of 52 gene clusters were identified in the draft genome of *A. ustus* 3.3904, such as the sterigmatocystin biosynthesis pathway that was commonly found in *Aspergillus* species. In addition, several SM biosynthetic gene clusters were firstly identified in *Aspergillus* that were possibly acquired by horizontal gene transfer, including the *vrt* cluster that is responsible for viridicatumtoxin production. Comparative genomics revealed that *A. ustus* shared the largest number of SM biosynthetic gene clusters with *A. nidulans*, but much fewer with other *Aspergilli* like *A. niger* and *A. oryzae*. These findings would help to understand the diversity and evolution of SM biosynthesis pathways in genus *Aspergillus*, and we hope they will also promote the development of fungal identification methodology in clinic.

## Introduction

Secondary metabolism in fungi produces a variety of chemical compounds including toxins and antibiotics. These secondary metabolites (SMs) are not necessary for fungus growth or conidiation, but sometimes confer pathogenicity, virulence and fungal adaptation to the environment [1,2]. To date, many secondary metabolism biosynthesis pathways (SMBPs) have been characterized in fungi. These pathways commonly contain ‘backbone enzymes’, *i.e.*, polyketide synthase (PKS), nonribosomal peptide synthetase (NRPS) or dimethylallyl transferase (DMAT), for generation of the carbon skeleton. And they also encode several other kinds of enzymes that perform post-modification of the intermediate products, *e.g.*, hydroxylase, O-methyltransferase and cytochrome P450. Genes involved in the biosynthesis of a particular metabolite are always organized in clusters and are often co-regulated, *e.g.*, the biosynthetic pathway for the well-known metabolites aflatoxin (AF) and sterigmatocystin (ST) contains about 26 genes, including a cluster specific transcription factor AflR that positively regulates the expression of about half the genes in the cluster [3–5].

Based on these common features of SM biosynthetic gene clusters, many bioinformatics tools have been developed and widely used to characterize the genetic basis of SM biosynthesis. These tools, such as the most frequently used SMURF and antiSMASH, are complementary to chemical and genetic approaches and have proven very useful in unraveling the biosynthesis pathways of novel compounds. Especially in the era of post genomics, fungal genome sequencing projects are rapidly increasing in number [6,7].

More than twenty *Aspergillus* genomes have been sequenced, including the main pathogens for invasive pulmonary aspergillosis (IPA) like *A. fumigatus, A. flavus* and *A. terrus*. However, the rarely found IPA pathogens, such as *A. alliaceus, A. ustus* and *A. udagawae* [8], have not been well studied at genome level. *A. ustus* belongs to the section *Usti* and is a mostly free living micro-organism. But it is also an opportunistic pathogen of immunocompromised patients, causing infection in lungs, eyes and hands [9–12]. However, in contrast to the extensive information available about other *Aspergillus* species, including *A. fumigatus* (the major cause of invasive pulmonary aspergillosis [13]), and *A. nidulans* (the model organism for studying a wide range of topics including development, cell cycle control and metabolism [14]), the biology and genetics of *A. ustus* is largely unknown. For a better understanding of the genetics of this rare pathogen, we sequenced the genome of a freeliving *A. ustus* strain and performed a comparative analysis with special attention to its SMBPs.

## Materials and Methods

### Genome sequencing and gene annotation

The *A. ustus* strain sequenced in this work, *A. ustus* 3.3904, was provided by the China General Microbiological Culture Collection Center (Beijing, China). Genomic DNA was prepared using the CTAB method as described previously [15]. Both a pair-end library (*ca*. 300 bp insertion) and a mate-pair library (*ca*. 3 kb insertion) were constructed and sequenced using HiSeq 2000 platform (Illumina, San Diego, CA, USA). Sequences were checked and assembled in the CLC Genomics Workbench (CLC Bio, Aarhus, Denmark). All reads were trimmed with default parameters and were then assembled by the *de novo* assembly module (word size 20). The draft assembled *A. ustus* genome has been deposited in GenBank under the accession number JOMC00000000.

Protein-coding genes in *A. ustus* were identified by AUGUSTUS software using the default parameters and the gene set of *A. nidulans* was used as the training data [16]. These predicted genes were primarily annotated by homology search against non-redundant protein database (National Center for Biotechnology Information, www.ncbi.nlm.nih.gov) using BLASTP tool (E-value<1e-3, identity>25%, query coverage>50%) [17].

The genome information of 21 sequenced *Aspergillus* species was downloaded from AspGD, including their predicted proteomes and the constructed orthologous groups (www.aspergillusgenome.org) (S1 Table). A comparative analysis of the predicted proteomes of *A. ustus* and the collected *Aspergillus* species was performed using BLASTP (E-value<1e-3, identity>25%, query coverage>50%). Gene ontology annotations were transferred to *A. ustus* genes when at least two of their best hits belonged to the same constructed orthologous group. Reciprocal best hits between *A. ustus* and *A. nidulans* were collected under strict parameters (E-value<1e-10, identity>50%, query coverage>50%).

### Identification of SM biosynthetic gene clusters

Identification of SM biosynthetic gene clusters in *A. ustus* 3.3904 was performed following these steps: Firstly, SM biosynthetic gene clusters in *A. ustus* were predicted by SMURF [18]. Secondly, these clusters were extended by three protein coding genes in genome on both sides and were then compared to the annotated clusters of *A. nidulans, A. niger* and *A. oryzae* using BLASTP (E-value<1e-10, identity>50%, query coverage>50%). If the newly added gene was mapped to the same genomic region as SMURF predicted cluster, or was assigned GO functions associated with the SM biosynthesis [19], it would be accepted as a component of the eventually determined *A. ustus* SMBPs and step2 would be repeated.

### Phylogenetic analysis

Phylogeny of *Aspergillus* species was investigated by analyzing their conserved proteins, which were collected by parsing the result of homology search between *A. ustus* and other *Aspergillus* species (E<1e-10, identity>80%, and the aligned regions covered >80% of both query and subject sequences) (S2 Table). ClustalW2 was used to align the concatenated sequences of selected proteins [20], and the alignment was subsequently used in neighbor-joining tree construction by MEGA 6 [21]. The phylogenetic tree was tested by bootstrap analysis with 1,000 replicates.

### Identification of horizontally acquired clusters

Putative horizontal transfer of SM biosynthetic gene clusters to *A. ustus* were discovered by the strategy similar to HGTector [22], *i.e*. a gene cluster predicted to be horizontally acquired should meet the following criterion: firstly, the cluster was absent in the closely related *Aspergillus* species; secondly, the cluster was found in distant organisms and the homologous gene pair shared pronounced sequence similarity (identity>50%).

## Results and Discussion

### Genome assembly and annotation

A total of 65 Mbp short reads were generated by sequencing the pair-end and mate-pair libraries. Approximately 94% of the short reads were assembled into 770 scaffolds (>500 bp) that represented the *A. ustus* 3.3904 chromosomes, with an average G+C content of 50.5%, a total length of 38.3 Mbp and an average length of 50 kbp. The chromosome-encoded genes were predicted by an *ab initio* method and by comparative genomics, which yielded a total of 13,143 predicted genes, showing a comparable gene density with other *Aspergillus* [23] (Table 1). Homologs for 93% of *A. ustus* protein coding genes in nr database, with about 75% genes had their best hits in other *Aspergillus* species.

**Table 1:** General features of *Aspergillus ustus* genome sequencing and annotation.

Features	Value
total reads	65M
average length of short reads	95 bp
No. of scaffolds (>500bp)	770
average length of scaffolds	49.8 kbp
length of the largest scaffold	1.03 Mbp
total length of scaffolds	38.35 Mbp
average G+C content (%)	50.5
No. of predicted coding genes	13,143

### Comparison of predicted proteomes

**Fig 1:**
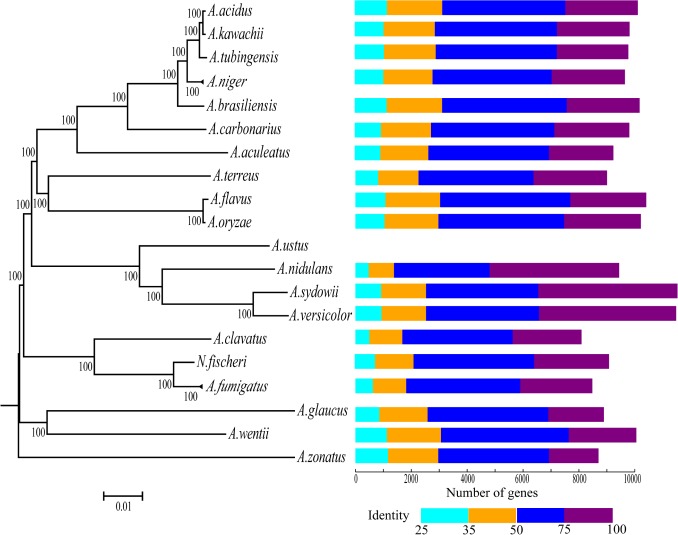
Comparative proteomes across *Aspergillus* species. The phylogenetic tree was constructed based on 375 highly conserved proteins in the *Aspergillus* genus. The right section shows the number of homologous genes between *A. ustus* and other *Aspergillus* species, with the color indicating the sequence identity derived from BLASTP (cutoff E-value 1e-3, identity 25% and query coverage 50%).

A phylogenetic analysis using several housekeeping genes, including beta-tubulin and cytochrome oxidase subunit I (*cox1*), has revealed the close relationship between *A. ustus* and the *Nidulantes* section species *A. nidulans* (which is also referred to as *Emericella nidulans* for its sexual form) [24]. In this study, a comparative analysis of the predicted proteomes was performed between *A. ustus* 3.3904 and the other 21 *Aspergillus* species, showing that 69% and 89% of the proteins encoded by *A. niger* (strain CBS 513.88) and *A. clavatus* (strain NRRL 1) (cutoff identity 25% and query coverage 50%), respectively, had homologs in *A. ustus*, with the other species scattered in between. Interestingly, further analysis found that *A. nidulans, A. sydowii* and *A. versicolor* shared a large number of highly similar genes (identity>75%) with *A. ustus*, which is consistent with their close relationships in phylogeny revealed by conserved genome content (Fig. 1).

*A. nidulans*, the species most closely related to *A. ustus* 3.3904 among investigated *Aspergilli*, has been extensively studied for SM biosynthesis, and its genome has been well annotated [14]. A comparative analysis between *A. ustus* and *A. nidulans* revealed a pronounced similarity between them in gene content that 70% of *A. nidulans* gene set had reciprocal best hits (RBH) in *A. ustus*. A detailed comparison of their SM biosynthesis was subsequently performed with the purpose of identifying the recent evolution of the SMBPs (the results are described in the next sections).

### Identification of SM biosynthetic gene clusters in *A. ustus*

SM biosynthetic gene clusters have been well annotated in genome-wide studies of some *Aspergillus* species, and the numbers vary greatly, for example, 81 for *A. niger* but only 39 for *A. fumigatus* [19]. In this study, a total of 52 SM biosynthetic gene clusters and partial clusters were predicted in *A. ustus* 3.3904 by bioinformatics analysis and comparative analysis (Table 2). More than half of these clusters contained genes encoding PKS/PKS-like enzymes, while the others encoding NRPS/NRPS-like (18) or DMAT (6), for generating the SM carbon skeletons. Although the products of most clusters in *A. ustus* remain unknown, some of them were predicted from the conservation of the SMBP components in the genus *Aspergillus*, including cluster14 (monodictyphenone), cluster39 (sterigmatocystin)(ST), cluster47 (emericellamide), cluster49 (ferricrocin) and cluster50 (asperthecin).

**Table 2:** Secondary metabolites biosynthetic gene clusters in *Aspergillus ustus*.

Cluster	Scaffold	Backbone enzymes	Genes	Predicted products
cluster1	132	DMAT	AUSM1_001–017	
cluster2	142	NRPS-Like	AUSM2_001–007	
cluster3	144	PKS	AUSM3_001–020	
cluster4	146	NRPS-Like	AUSM4_001–015	
cluster5	154	NRPS	AUSM5_001–009	
cluster6	155	PKS	AUSM6_001–028	
cluster7	156	DMAT	AUSM7_001–004	
cluster8	200	PKS	AUSM8_001–040	
cluster9	202	NRPS-Like	AUSM9_001–005	
cluster10	212	PKS	AUSM10_001–014	
cluster11	15	NRPS-Like	AUSM11_001–017	
cluster12	229	DMAT	AUSM12_001–015	
cluster13	230	PKS	AUSM13_001–009	
cluster14	253	PKS	AUSM14_001–017	monodictyphenone
cluster15	16	PKS	AUSM15_001–008	
cluster16	17	PKS	AUSM16_001–005	
cluster17	18	PKS	AUSM17_001–007	
cluster18	19	PKS	AUSM18_001–008	
cluster19	21	NRPS	AUSM19_001–007	
cluster20	21	DMAT	AUSM20_001–014	
cluster21	2	PKS-Like	AUSM21_001–007	
cluster22	25	NRPS-Like	AUSM22_001–005	
cluster23	25	NRPS-Like	AUSM23_001–017	
cluster24	26	PKS	AUSM24_001–022	
cluster25	28	PKS	AUSM25_001–015	
cluster26	31	PKS	AUSM26_001–017	
cluster27	33	PKS	AUSM27_001–019	viridicatumtoxin
cluster28	33	NRPS-Like	AUSM28_001–018	
cluster29	36	PKS	AUSM29_001–028	
cluster30	36	PKS	AUSM30_001–022	
cluster31	39	PKS	AUSM31_001–008	
cluster32	43 & 37 ^a^	PKS	AUSM32_001–014	
cluster33	43	NRPS-Like	AUSM33_001–022	
cluster34	43	DMAT	AUSM34_001–011	
cluster35	48	NRPS	AUSM35_001–009	
cluster36	58	NRPS	AUSM36_001–013	
cluster37	58	NRPS-Like	AUSM37_001–011	
cluster38	63	PKS	AUSM38_001–022	
cluster39	65	PKS-Like	AUSM39_001–026	sterigmatocystin
cluster40	65	PKS	AUSM40_001–025	
cluster41	68	PKS	AUSM41_001–005	
cluster42	69	PKS	AUSM42_001–007	
cluster43	70	NRPS-Like	AUSM43_001–007	
cluster44	8	PKS	AUSM44_001–021	F-9775A
cluster45	79	PKS	AUSM45_001–018	
cluster46	88	PKS	AUSM46_001–026	
cluster47	103	NRPS	AUSM47_001–011	emericellamide
cluster48	104	NRPS	AUSM48_001–007	
cluster49	105	NRPS	AUSM49_001–005	ferricrocin
cluster50	114	PKS	AUSM50_001–003	asperthecin
cluster51	121	DMAT	AUSM51_001–006	
cluster52	121	NRPS	AUSM52_001–003	

a. Two partial of such secondary biosynthetic gene cluster locate in the ends of scaffolds 43 and 37 of *Aspergillus ustus* draft assembly, respectively.

Moreover, some SMBPs in *A. ustus* 3.3904 were characterized in *Aspergillus* species for the first time, such as the biosynthesis pathway of viridicatumtoxin (VRT) (cluster27). VRT is a tetracycline-type antibiotic, which can inhibit the growth of methicillin-resistant and quinolone-resistant *Staphylococcus aureus* with high activity [25]. Until recently, the *vrt* cluster was only characterized in *Penicillium aethiopicum*, including *vrtA-L* and two regulators (*vrtR1* and *vrtR2*), which was assumed to be a recent acquisition by horizontal gene transfer [26]. In this study, homology search of this cluster against nr database identified another *vrt* cluster that was encoded by the genome of an insect pathogen, *Metarhizium acridum* [27] (BLASTP with the cutoff identity 50% and query coverage 50%). And in *A. ustus* 3.3904, we found a *vrt* gene cluster containing 19 genes (AUSM27_001–019), most of which shared the sequence identities of 56–76% and 62–71% with their counterparts in *P. aethiopicum* and *M. acridum*, respectively. The possible regulatory components in *A. ustus vrt* cluster diverged much more than the other components, *i.e.*, the *vrtR1* gene was found not embedded in the *vrt* gene cluster (*ca*. 55% identity), and the VrtR2 showed low sequence identity (*ca*. 36%) to its orthologs. Notably, the *vrt* cluster of *A. ustus* 3.3904 is lack of the coding genes of VrtE (cytochrome P450) and VrtF (O-methyltransferase), thus further study is necessary to determine the end products.

Some organic compounds produced by another *A. ustus* strain, KMM 4640, have been determined by chemical methods, including patulin (PAT), ST and cladosporin [28]. Among them, PAT biosynthetic pathway has been characterized in some other *Aspergilli* and *Penicillia*, like *Aspergillus clavatus* and *Penicillium expansum* [29], however, a homology search in *A. ustus* 3.3904 did not find any potential gene cluster encoding enzymes associated with PAT biosynthesis (cutoff E-value<1e-3, identity>25% and query coverage>50%), indicating that production of PAT is not a common feature of *A. ustus*.

### Comparative analysis of SM biosynthetic gene clusters in *Aspergillus* species

The increasing availability of *Aspergillus* genomes has led to a rapid identification of SMBPs in recent years and subsequently has revealed that only a small proportion of SMBPs are conserved between even closely related species [19]. In this work, comparative analysis of SM biosynthetic gene clusters was performed between *A. ustus* and the other three well annotated *Aspergillus* species, *i.e., A. nidulans, A. niger* and *A. oryzae*, revealing that *A. ustus* shared the greatest number of SMBPs with *A. nidulans*, like cluster2 (*ivo*), cluster18 (*pki*), cluster 32(*pkf*) and several other gene clusters with function unknown (S3 Table). Much less SM biosynthetic gene clusters were conserved in *A. niger* or *A. oryzae*, but interestingly, the cluster35 (function unknown) was highly conserved among all these four species. We also checked the *A. ustus* SM biosynthetic gene clusters in other sequenced *Aspergillus* species and found five of them were specific to *A. ustus*, which therefore indicated horizontal acquisition. Similar gene clusters for four of them, *i.e.*, cluster 27, 29, 44 and 45, were found in *P. aethiopicum, Trichoderma virens* or *Talaromyces marneffei* (S4 Table), indicating penicillia are important reservoir of SM biosynthetic gene clusters in *A. ustus*.

**Fig 2:**
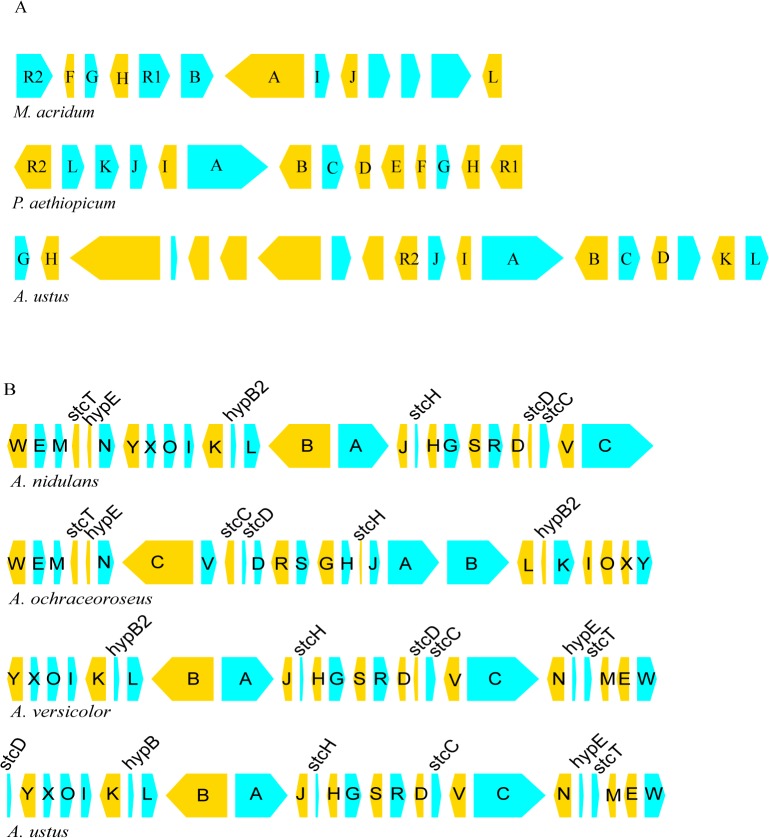
Arrangement of the *vrt* and *st* gene clusters in *Aspergillus ustus* and closely related species. vrt: viridicatumtoxin. st: sterigmatocystin. The arrow indicates the coding strand, the width of boxes and spaces were drawn in scale according to the genome annotation.

In addition, the gene organization of particular SM clusters in different fungi also shows remarkable variation. Although the gene organization does not appear to be crucial for SM biosynthesis [30], it would provide another way to understand fungal evolution. As most of the SMBP components have not been well characterized, the *st* cluster that was usually found in *Aspergillus* as well as the *vrt* cluster, which was found in *Aspergillus* for the first time, were used, for example, to show the variation of the organization in SM clusters (Fig. 2).

The *st* clusters of *A. ustus* and another three closely related species (*A. versicolor, A. nidulans* and *A. ochraceoroseus*) were compared. The contents of the *st* clusters in these species are almost the same, and the *st* genes share amino acid identities of 46–87% between *A. ustus* and the other species. Interestingly, the structure of the *st* cluster in *A. ustus* was most like that of *A. versicolor* rather than like that of *A. nidulans*, with a slight variation in the *stcD* location, *i.e.*, in the other three *Aspergillus* species, *stcC* and *stcD* are commonly coupled and located between *aflV* and *aflD*, with the structure *aflV*-*stcC*-*stcD*-*aflD*. However, in *A. ustus*, only *stcC* was found between *aflV* and *aflD*, while *stcD* was located at the end of the *st* cluster.

In contrast, the variation in the organization of the *vrt* clusters in *A. ustus, P. aethiopicum* and *M. acridum* is remarkable in that only small scaled conservation was found, such as *vrtJ*-*vrtI*-*vrtA*-*vrtB*. In addition, the *vrt* cluster in *A. ustus* contains seven additional genes between *vrtH* and *vrtR2*, including two PKS-like genes that share *ca*. 60% identity with their homologs in *M. acridum*. Notably, in *M. acridum*, orthologs for these two PKS-like genes (EFY92556 and EFY92553) and another laccase encoded gene (EFY92552) are also clustered on chromosome. The flanking genomic regions of the *A. ustus vrt* cluster were analyzed, but there was no syntenic region found in the genomes of other *Aspergillus* species. These findings indicated that the *vrt* cluster in *A. ustus* might result from two independent evolutionary events, including a remote insertion into the *vrt* cluster of a segment, which possibly occurred in an organism other than a member of the genus *Aspergillus*, and a subsequent HGT of the newly formed *vrt* cluster into *A. ustus*, which very possibly took place after the divergence of *A. ustus* from the other *Aspergilli*.

## Concluding Remarks and Perspective

An increasing amount of knowledge about human fungal pathogens is becoming available, largely derived from genome sequencing projects. In this study, we sequenced and annotated the genome of a rare human pathogen, *A. ustus* and revealed the phylogenetic relationship between *A. ustus* and other *Aspergilli* at the whole-genome level. Interestingly, although *A. ustus* and *A. nidulans* are the most closely related species and share a large number of highly similar genes, their genome size varied a lot, *i.e.*, 38M and 30M, respectively. This is partially caused by the incomplete state of *A. ustus* genome, but also indicates large scaled gene gain/loss in recent adaptation evolution. SMBPs were systematically analyzed in *A. ustus* 3.3904 and 52 SM gene clusters were found, including the *vrt* cluster that was found in *Aspergillus* for the first time. In addition, extensive differences in the SMBPs were also identified, from the products profile to the gene content and organization in particular SM clusters, showing that SM biosynthesis is under rapid evolution in *Aspergillus*, and might be associated with the complex environment they confront.

Invasive fungal infections, including IPA, plagues a large number of immunocompromised patients. The early diagnosis of IPA is of great benefit to prognosis; however, it is still a challenge for the current methods used in clinics. Some of the methods are time consuming (*e.g.*, blood or sputum culture) or lacking in both sensitivity and specificity (*e.g.*, computed tomography (CT) and the beta-D-glucan/galactomannan test). Therefore, new molecular methods, such as microarray and PCR assays, are anticipated to provide higher sensitivity and speed. For example, PCR based methods have been greatly improved by the efforts of EAPCRI (Working Group European *Aspergillus* PCR Initiative), which aims to develop a standard for *Aspergillus* PCR methodology (www.eapcri.eu), and a recent study has revealed that combined sequencing of multiple genomic sites would improve the performance [31]. In this sense, the generation of more *Aspergillus* genome information remains important and would be of great help in screening for new biomarkers for *Aspergillus* identification in clinics.

## Supporting Information

S1 Table The *Aspergillus* species investigated in the comparative analysis of this work. (XLSX)

S2 Table Conserved proteins among *Aspergillus* species. (XLSX)

S3 Table Homologs of secondary metabolites biosynthesis associated genes between *Aspergillus ustus* and the closely related *A. nidulans, A. niger* and *A. oryzae*. (XLSX)

S4 Table Putative horizontally acquired gene clusters and homologs of their encoded proteins. (XLSX)

## References

[1] Fox EM, Howlett BJ (2008) Secondary metabolism: regulation and role in fungal biology. Curr Opin Microbiol 11: 481–487. 10.1016/j.mib.2008.10.007 18973828

[2] Osbourn A (2010) Secondary metabolic gene clusters: evolutionary toolkits for chemical innovation. Trends Genet 26: 449–457. 10.1016/j.tig.2010.07.001 20739089

[3] Brown DW, Yu JH, Kelkar HS, Fernandes M, Nesbitt TC, et al (1996) Twenty-five coregulated transcripts define a sterigmatocystin gene cluster in Aspergillus nidulans. Proc Natl Acad Sci U S A 93: 1418–1422. 8643646 10.1073/pnas.93.4.1418 PMC39953

[4] Ehrlich KC, Montalbano BG, Cary JW (1999) Binding of the C6-zinc cluster protein, AFLR, to the promoters of aflatoxin pathway biosynthesis genes in Aspergillus parasiticus. Gene 230: 249–257. 10216264 10.1016/s0378-1119(99)00075-x

[5] Fernandes M, Keller NP, Adams TH (1998) Sequence-specific binding by Aspergillus nidulans AflR, a C6 zinc cluster protein regulating mycotoxin biosynthesis. Mol Microbiol 28: 1355–1365. 9680223 10.1046/j.1365-2958.1998.00907.x

[6] Fedorova ND, Moktali V, Medema MH (2012) Bioinformatics approaches and software for detection of secondary metabolic gene clusters. Methods Mol Biol 944: 23–45. 10.1007/978-1-62703-122-6_2 23065606

[7] Weber T (2014) In silico tools for the analysis of antibiotic biosynthetic pathways. Int J Med Microbiol 304: 230–235. 10.1016/j.ijmm.2014.02.001 24631213

[8] Balajee SA, Houbraken J, Verweij PE, Hong SB, Yaghuchi T, et al (2007) Aspergillus species identification in the clinical setting. Stud Mycol 59: 39–46. 10.3114/sim.2007.59.05 18490954 PMC2275201

[9] Olorunnipa O, Zhang AY, Curtin CM (2010) Invasive Aspergillosis of the Hand Caused by Aspergillus ustus: a Case Report. Hand (N Y) 5: 102–105. 19568818 10.1007/s11552-009-9211-x PMC2820612

[10] Cabada MM, Nishi SP, Lea AS, Schnadig V, Lombard GA, et al (2010) Concomitant pulmonary infection with Nocardia transvalensis and Aspergillus ustus in lung transplantation. J Heart Lung Transplant 29: 900–903. 10.1016/j.healun.2010.04.016 20541440

[11] Florescu DF, Iwen PC, Hill LA, Dumitru I, Quader MA, et al (2009) Cerebral aspergillosis caused by Aspergillus ustus following orthotopic heart transplantation: case report and review of the literature. Clin Transplant 23: 116–120. 10.1111/j.1399-0012.2008.00895.x 18798849

[12] Vagefi PA, Cosimi AB, Ginns LC, Kotton CN (2008) Cutaneous Aspergillus ustus in a lung transplant recipient: emergence of a new opportunistic fungal pathogen. J Heart Lung Transplant 27: 131–134. 10.1016/j.healun.2007.09.020 18187099

[13] Nierman WC, Pain A, Anderson MJ, Wortman JR, Kim HS, et al (2005) Genomic sequence of the pathogenic and allergenic filamentous fungus Aspergillus fumigatus. Nature 438: 1151–1156. 16372009 10.1038/nature04332

[14] Galagan JE, Calvo SE, Cuomo C, Ma LJ, Wortman JR, et al (2005) Sequencing of Aspergillus nidulans and comparative analysis with A. fumigatus and A. oryzae. Nature 438: 1105–1115. 16372000 10.1038/nature04341

[15] Sun X, Ruan R, Lin L, Zhu C, Zhang T, et al (2013) Genomewide investigation into DNA elements and ABC transporters involved in imazalil resistance in Penicillium digitatum. FEMS Microbiol Lett 348: 11–18. 10.1111/1574-6968.12235 23952944

[16] Stanke M, Steinkamp R, Waack S, Morgenstern B (2004) AUGUSTUS: a web server for gene finding in eukaryotes. Nucleic Acids Res 32: W309–312. 15215400 10.1093/nar/gkh379 PMC441517

[17] Altschul SF, Madden TL, Schaffer AA, Zhang J, Zhang Z, et al (1997) Gapped BLAST and PSI-BLAST: a new generation of protein database search programs. Nucleic Acids Res 25: 3389–3402. 9254694 10.1093/nar/25.17.3389 PMC146917

[18] Khaldi N, Seifuddin FT, Turner G, Haft D, Nierman WC, et al (2010) SMURF: Genomic mapping of fungal secondary metabolite clusters. Fungal Genet Biol 47: 736–741. 10.1016/j.fgb.2010.06.003 20554054 PMC2916752

[19] Inglis DO, Binkley J, Skrzypek MS, Arnaud MB, Cerqueira GC, et al (2013) Comprehensive annotation of secondary metabolite biosynthetic genes and gene clusters of Aspergillus nidulans, A. fumigatus, A. niger and A. oryzae. BMC Microbiol 13: 91 10.1186/1471-2180-13-91 23617571 PMC3689640

[20] Larkin MA, Blackshields G, Brown NP, Chenna R, McGettigan PA, et al (2007) Clustal W and Clustal X version 2.0. Bioinformatics 23: 2947–2948. 17846036 10.1093/bioinformatics/btm404

[21] Tamura K, Stecher G, Peterson D, Filipski A, Kumar S (2013) MEGA6: Molecular Evolutionary Genetics Analysis version 6.0. Mol Biol Evol 30: 2725–2729. 10.1093/molbev/mst197 24132122 PMC3840312

[22] Zhu Q, Kosoy M, Dittmar K (2014) HGTector: an automated method facilitating genome-wide discovery of putative horizontal gene transfers. BMC Genomics 15: 717 10.1186/1471-2164-15-717 25159222 PMC4155097

[23] Sanchez JF, Somoza AD, Keller NP, Wang CC (2012) Advances in Aspergillus secondary metabolite research in the post-genomic era. Nat Prod Rep 29: 351–371. 10.1039/c2np00084a 22228366 PMC4568942

[24] Krimitzas A, Pyrri I, Kouvelis VN, Kapsanaki-Gotsi E, Typas MA (2013) A phylogenetic analysis of Greek isolates of Aspergillus species based on morphology and nuclear and mitochondrial gene sequences. Biomed Res Int 2013: 260395 10.1155/2013/260395 23762830 PMC3665174

[25] Zheng CJ, Yu HE, Kim EH, Kim WG (2008) Viridicatumtoxin B, a new anti-MRSA agent from Penicillium sp. FR11. J Antibiot (Tokyo) 61: 633–637. 10.1038/ja.2008.84 19168978

[26] Chooi YH, Cacho R, Tang Y (2010) Identification of the viridicatumtoxin and griseofulvin gene clusters from Penicillium aethiopicum. Chem Biol 17: 483–494. 10.1016/j.chembiol.2010.03.015 20534346 PMC2884005

[27] Gao Q, Jin K, Ying SH, Zhang Y, Xiao G, et al (2011) Genome sequencing and comparative transcriptomics of the model entomopathogenic fungi Metarhizium anisopliae and M. acridum. PLoS Genet 7: e1001264 10.1371/journal.pgen.1001264 21253567 PMC3017113

[28] Oleinikova GK, Denisenko VA, Slinkina NN, Afiyatullov SS (2012) Secondary metabolites of the marine fungus Aspergillus ustus KMM 4640. Chemistry of Natural Compounds 48: 467–469.

[29] Artigot MP, Loiseau N, Laffitte J, Mas-Reguieg L, Tadrist S, et al (2009) Molecular cloning and functional characterization of two CYP619 cytochrome P450s involved in biosynthesis of patulin in Aspergillus clavatus. Microbiology 155: 1738–1747. 10.1099/mic.0.024836-0 19383676 PMC2889413

[30] Palmer JM, Keller NP (2010) Secondary metabolism in fungi: does chromosomal location matter? Curr Opin Microbiol 13: 431–436. 10.1016/j.mib.2010.04.008 20627806 PMC2922032

[31] Wang X, Fu YF, Wang RY, Li L, Cao YH, et al (2014) Identification of clinically relevant fungi and prototheca species by rRNA gene sequencing and multilocus PCR coupled with electrospray ionization mass spectrometry. PLoS One 9: e98110 10.1371/journal.pone.0098110 24835205 PMC4024029

